# Epigenetic Changes Associated with Different Types of Stressors and Suicide

**DOI:** 10.3390/cells12091258

**Published:** 2023-04-26

**Authors:** Garrett Dee, Rebecca Ryznar, Colton Dee

**Affiliations:** 1 College of Osteopathic Medicine, Rocky Vista University, Parker, CO 80112, USA; 2 Molecular Biology, Department of Biomedical Sciences, Rocky Vista University, Parker, CO 80112, USA; 3 College of Osteopathic Medicine, Des Moines University, Des Moines, IA 50312, USA

**Keywords:** epigenetic, DNA methylation, microRNA, histone modification, stress, acute stress, chronic stress, early childhood stress, traumatic stress, suicide

## Abstract

Stress is associated with various epigenetic changes. Some stress-induced epigenetic changes are highly dynamic, whereas others are associated with lasting marks on the epigenome. In our study, a comprehensive narrative review of the literature was performed by investigating the epigenetic changes that occur with acute stress, chronic stress, early childhood stress, and traumatic stress exposures, along with examining those observed in post-mortem brains or blood samples of suicide completers and attempters. In addition, the transgenerational effects of these changes are reported. For all types of stress studies examined, the genes *Nr3c1*, *OXTR*, *SLC6A4*, and *BDNF* reproducibly showed epigenetic changes, with some modifications observed to be passed down to subsequent generations following stress exposures. The aforementioned genes are known to be involved in neuronal development and hormonal regulation and are all associated with susceptibility to mental health disorders including depression, anxiety, personality disorders, and PTSD (post-traumatic stress disorder). Further research is warranted in order to determine the scope of epigenetic actionable targets in individuals suffering from the long-lasting effects of stressful experiences.

## 1. Introduction

The field of epigenetics has gained greater appreciation in recent years. Determining the repertoire of epigenetic modifications that occur across the genome following stress can provide insight into not only the temporary effects of stress on the biology of an individual but can reveal valuable information about individualized future responses to stress. The term epigenetics is defined by Berger et al. as a “… stably heritable phenotype resulting from changes in a chromosome without alterations in the DNA sequence” [1]. It is well known that epigenetic changes are passed down somatically from cell to cell and in some cases transgenerationally, meaning the epigenetic change persists in subsequent generations, even in the absence of the stressor. Berger et al. further break down epigenetics into three categories which they label “Epigenators”, an environmental trigger that affects the cellular processes within the cell, “Initiators”, an intracellular signal that elicits an effect on the epigenome, and “Maintainers”, local histone variants and DNA methylation that maintain the chromatin state. The Epigenator is the upstream signal from the environment or another signaling pathway that activates the Initiator. An example of this is demonstrated by Cheesman and Weitzman in parasites from the apicomplexa phylum and their proteome acting as an Epigenator in their supposed pathogenesis [1,2]. The Initiator then interprets the signal for the precise location at which to act [1]. This location could include DNA, noncoding RNA, histones, and other structures involved in chromatin remodeling [1]. The Maintainer supports the “epigenetic chromatin state” at the specific location through DNA methylation and other specific epigenetic modifications [1]. 

Epigenetic modifications include DNA methylation, noncoding RNA such as microRNA, histone modification via methylation, acetylation, ubiquitination, and phosphorylation, and others including SUMOylating. DNA methylation works through enzymes known as DNA methyl transferases that transfer a methyl group to a cytosine-guanine dinucleotide (CpG) region in the DNA [3]. DNA methylation patterns correlate with gene expression levels [4], specifically a reduction in transcription. Histone modification can occur through either the methylation of an arginine or the acetylation of a lysine similar to DNA and can lead to chromosome condensing or opening [3]. Non-coding RNAs are RNA molecules that do not code for a protein but assist in other functions. An example of a non-coding RNA is microRNA (miRNA). miRNA is able to interact with genetic material such as mRNA in such a way as to control gene expression [5]. These epigenetic changes are subjected to a host of outside influences ranging from various types of stress, nutrition, lifestyle choices, and the environment [6,7]. The permanence of these markers is often a question of research and is still being explored. 

Healthy physiological responses to stress are designed to maintain homeostasis and survivability [7,8]. Once stress is detected, the hypothalamic-pituitary-adrenal (HPA) axis is activated by the paraventricular nucleus of the hypothalamus releasing corticotropin-releasing hormone (CTRH), which activates the anterior pituitary to release adrenocorticotropic hormone (ACTH). ACTH exerts its effect on the adrenal gland by releasing glucocorticoids such as cortisol that have a body-wide effect on locations that have glucocorticoid receptors (*Nr3c1*) and a negative effect on the hypothalamus and anterior pituitary, releasing their respective stress hormones. When stress is encountered, it modifies the rhythmic pulsatile nature of the release of CTRH by increasing it [7]. Stress has also been shown to affect the immune system [9]. As reviewed by Dragos et al., acute stress has been shown to increase the resistance to infection whereas chronic stress impairs it [10]. Additionally, a study conducted on acute traumatic brain injury individuals showed an inverse correlation between circulating immune T cells and plasma cortisol levels in mice [11]. 

Stress is an inevitable part of life. In this review, we focus on five categories of different types of stress and their associated epigenetic changes. Depending on the type of stress experienced by an individual, stress can act as the epigenator in human cells, resulting in initiator-induced changes that can result in stable epigenetic changes through the activity of maintainers. Different types of stress epigenators include acute stress, chronic stress, early childhood stress, traumatic stress, and suicide. Acute stress is considered as an event that is brief in nature or mimics an acute event that causes stress on a subject. Chronic stress is defined as an event that occurs over a longer period of time or an experiment that mimics a chronic stressor on a subject. A stressful event occurring during adolescence is considered as early childhood stress but does not include in utero maternal stress. Traumatic stress is defined as an event that had some sort of terrifying, dangerous, and or shocking impact [12,13] leading to either acute stress disorder or post-traumatic stress disorder (PTSD) or is highly correlated with the development of these disorders following exposure [14]. Suicide is defined as either the thought of ending one’s life, attempting suicide, or completing the act.

This review incorporates the different aspects of stressors and suicide with their relation to epigenetics in one complete narrative review. We explore some of the epigenetic changes that are reported following different types of stress such as acute, chronic, early childhood, and traumatic, along with suicide contemplation/completion. We ask the question, what are the commonalities between the different epigenetic changes caused by the different forms of stress? Do different stressors affect the same genes or genomic regions? Are these epigenetic changes inherited in a transgenerational fashion? What are the implications of these findings with regard to molecular psychiatry and pharmacological treatments?

## 2. Acute Stress

Acute stress is defined as an event that inflicts stress on the subject for a short period of time before removing it. In the literature, to evaluate the effects of acute stress, researchers tested it in a multitude of ways ranging from acute exposure of benzo[a]pyrene and UV light, restraint tests, heat stress, trier social stress test (TSST), psychosocial stress, and swim tests. Studies assessing epigenetic changes associated with acute stress have been conducted in mice, chickens, rats, clams, and humans. In an experiment with blood clam gills, Guo et al. demonstrated that the acute exposure to benzo[a]pyrene, a compound found in tobacco smoke, coal, and tar, resulted in a decrease in global DNA methylation levels [15], suggesting brief exposure to chemical stressors such as benzo[a]pyrene could potentially lead to epigenetic changes in the model organism. In addition to these findings, the literature has recently demonstrated that immune cell portions (lymphocytes) can be a potential estimator for whole-genome DNA methylation [16]. In a study conducted on adult humans with an acute psychosocial stressor, Apsley et al. showed an increase in immune cells and a concordant increase in whole genome DNA methylation changes [16]. 

An important receptor in the stress response is the glucocorticoid receptor. Without it, the model organism (i.e., mice and humans) would not be able to elicit a full response to stress due to a lack of receptor binding. This could potentially lead to disorders of the brain including depression [17]. Various studies have looked at the methylation changes in different regions of the glucocorticoid receptor gene (*Nr3c1*). Using mice as a model system, Li et al., evaluated the 3′UTR region of *Nr3c1* 5-hydroxymethylcytosine (5-hmC) changes due to an acute restraint stress test [18,19]. They discovered an increase in 5-hmC in the 3′UTR region due to stress in the hippocampus of male mice. The second group of scientists, Rooij et al., looked at the methylation in the 1-C promoter region in the *Nr3c1* gene of (N = 675) human participants due to three different psychosocial stressors such as a speech test, mirror-tracing tests, and a Stroop test [20]. They observed that lower stress reactivity measured through heart rate and cortisol response was associated with a lower level of methylation in the 1-C promoter region of fasting blood samples. However, when these results were adjusted for lifestyle variables such as sex, smoking, etc., the associations dissolved suggesting that lifestyle differences play a larger role. Interestingly, the lower methylation levels found in promoter region 1-C of the *Nr3c1* gene were associated with a higher perception of stress and a decrease in perceived control and performance. The third group of scientists, Mifsud et al., focused on an area upstream of exon 2 in the *Nr3c1* gene called “GR area 1” and a region around exon 1_7_ called “GR area 2” in male Wistar rats’ hippocampi [21]. The CpG levels of “GR area 1” were not affected in dentate gyrus or Cornu Ammonis, but “GR area 2” showed a significant increase in methylation in dentate gyrus and a decrease in Cornu Ammonis due to a swim stress test. An examination of epigenetic changes along the *Nr3c1* gene between human and animal studies showed variable region changes reflective of the different acute stressors applied.

Recent literature has assessed chromatin shape changes associated with acute stress, specifically histone methylation and acetylation. Hunter et al. looked at histone H3 lysine 3 (H3K9) modifications at retro-transposable element loci in the hippocampi of male Sprague Dawley rats [22]. They discovered that acute stress was associated with H3K9me3 and that the H3K9 methyl transferase, Suv39h2, is up-regulated in the hippocampus in acute restraint stress groups. In another study focusing on histone modification, Zheng et al. looked at Histone H3 lysine 27 (H3K27) methylation changes due to acute heat stress in (N = 192) Taiwan country chickens [23]. They discovered that chickens whose body temperature changed more than 6.5 °C (the susceptible group) had an increase in H3K27me3 compared to controls in addition to showing positive crosstalk with K36me and K37me in the tails of H3. When looking at the dentate gyrus and CA1 region of the hippocampus in adult male Sprague Dawley rats, Hunter et al.’s prior study examined histone (H3K3, H3K9, and H3K27) modifications [24]. The researchers determined that acute restraint stress was associated with an increase in H3K9me3 in both the dentate gyrus and CA1, whereas there was no effect on H3K4me3. They also observed a reduction in both H3K9me1 and H3K27me3 in the same regions. When comparing these results across the board, there is a consistent me3 in the H3 histone in animal models with no human study to compare to. In the last study focusing on histone modification, Ieraci et al. looked at histone H3 in the promoter region of the brain-derived neurotrophic factor (BDNF) of male mice after an acute restraint stress test [25]. Their results showed no change in the methylation or acetylation status of H3. Interestingly, they found a decrease in the mRNA levels of BDNF after the acute restraint stress test suggesting that there may be another epigenetic mechanism that regulates BDNF expression.

When turning our focus to miRNA (noncoding RNA) affecting gene expression under acute stress conditions, two literature articles were unearthed that report the involvement of specific miRNAs. The first article, which was previously discussed under *Nr3c1* by Mifsud et al., also addressed miR-124a, a potential regulator of the *Nr3c1* mRNA, and its association with the swim stress test [21]. They observed an increase in expression after a forced swim test in a time-dependent manner while also noticing a decrease in *Nr3c1* mRNA levels in the dentate gyrus. Mifsud et al. hypothesize that this may offer a protective mechanism in response to acute stress in the dentate gyrus to repeated forms of stress. Mannironi et al. examined two different miRNAs, miR-135a and miR-124, in adult male mice amygdala after an acute restraint test in the context of acute stress [26]. They determined that in the acute stress response, these two miRNAs are down-regulated, which correlated with higher expression levels of another stress response receptor, the mineralocorticoid receptor, in the amygdala.

Additionally, various studies reported epigenetic changes to genes associated with the regulation of vitamin D levels, tumor suppressors, structural framework, immune response, and oxytocin regulation, associated with acute stress. These genes that encode for different types of proteins included *CYP24A1*, *BRCA2*, *NOTCH2*, *FOXO3*, *GATA3*, *CSNK2A2*, *KRT17*, *CARD14*, *IRF8*, *BDNF*, *OXTR*, and *PRF1*. In a study performed on (N = 32) healthy White females of different Fitzpatrick phototypes (a scale used to predict sunburn risk) by applying acute exposure of UV light (which is most likely different compared to psychological stress) to tissue from the sun-protected area of the lower back [27]. They observed *CYP24A1*, *BRCA2*, *NOTCH2*, *FOXO3*, and *GATA3* to be methylated, *KRT17* and *CSNK2A2* to be hypomethylated, *CARD14* to be demethylated, and *IRF8* to be hypermethylated upon acute exposure to UV radiation. Unternaehrer et al. examined two regions in the oxytocin receptor gene (*OXTR*) and the exon V, Vh, and Vi in the *BDNF* of human blood samples that underwent the TSST [28]. They found no associated differences in *BDNF* methylation in the human blood samples but did find an increase in methylation from pre- to post-stress event in the *OXTR* exon III protein coding region (“*OXTR1*”) and a decrease in methylation from post-stress to follow up in the OXTR exon III noncoding/coding promoter region (“*OXTR2*”). These results remained significant even after the blood cell count was controlled. Another study that conducted the TSST but in chronic fatigue syndrome patients observed epigenetic changes in the promoter region of the perforin protein (*PRF 1*) that creates a channel in cell membranes during an immune response [29]. These epigenetic changes showed an increase in methylation in two *PRF 1* CpG sites, -776 and -774, after the TSST in chronic fatigue syndrome blood sampling; however, there were no significant differences between the chronic and non-chronic fatigue syndrome groups. 

Lastly, a group of researchers, Li et al., observed the down-regulation of gene expression in the hippocampus of a variety of different genes coding for phosphodiesterase/lipase, a bone morphogenetic protein antagonist, proteins involved with neuronal development, and secreted signaling proteins such as *Enpp2*, *Sostdc1*, *Ulk4*, and *Wnt9a* after an acute restraint test was performed on mice [19]. *Enpp2* and *Sostdc1* were hyper-differentially hydroxymethylated (DhMR) in the intron region and the upstream region of the transcription start site, respectively. *Ulk4* and *Wnt9a* were hypo-DhMR in the upstream region of the transcription start site and intron/exon region, respectively. Li et al. also demonstrated up-regulation of genes involved in sequestering p53, transcription suppression and activation, the regulation of growth and apoptosis, cytoplasmic signaling molecules for insulin, structural proteins, and neuronal development such as *Banp*, *Cbfa2t3*, *Gadd45b*, *Irs2*, *Klf15*, *Smtn*, and *Spns2* due to the same stress on mice [19]. All of these genes were observed to be hypo-DhMR. *Banp* and *Gadd45b* were observed to be hypo-DhMR downstream of the genes, whereas *Cbfa2t3* was modified upstream of the transcription start site. The *Irs2* and *Spns2* hypo-DhMR region was reported in the exon and intron regions. *Klf15* and *Smtn* were observed to be hypo-DhMR along the span of the gene sequence, including intronic and exonic regions. Overall, acute stress was shown to cause a variety of epigenetic changes in a diverse set of genes. The overall summary of these epigenetic changes associated with acute stress can be seen in Table 1.

## 3. Chronic Stress

Chronic stress is defined as exposure to a reoccurring stressor over an extended period of time. In animal models, a chronic restraint test, a forced swim test in varying water temperatures over consecutive days, chronic water avoidance, exposure to other animals, and social defeat tests are common modalities of chronic stress tests. In human subjects, chronic stress effects are gleaned from participants being exposed to chronic job stress, living situations, elevated platforms, and chronic social defeat tests. Studies assessing epigenetic changes during chronic stress have been conducted and observed in mice, rats, and humans. In McEwen’s review on the system-wide effects of chronic stress, he highlights that chronic stress can have gross changes in the brain resulting in neuronal imbalance [30]. These large-scale modifications can also lead to behavioral changes [31]. A potential cause of this imbalance could be the influence of epigenetics when looking at the gene x environment model. 

The Nr3c1 receptor plays a vital role in the stress response for both acute and chronic stress. In the literature, chronic stress has been shown to down-regulate the glucocorticoid receptor mRNA expression levels in the brain [32,33]. In particular, Louwies et al. investigated epigenetic changes in the *Nr3c1* gene promoter region of exon 1_7_ in (N = 36) female Fischer-344 rats [34]. They observed increases in DNA methylation in the central nucleus of the amygdala with 7 days of consecutive chronic water avoidance stress. Previously, they saw the down-regulation of the Nr3c1 receptor expression in the same tissue due to the same stressor [35]. Another group of scientists, Witzmann et al., also examined the promoter region of exon 1_7_ but in male Sprague Dawley rats’ (N = 24 for chronic group) adrenal and pituitary glands [36]. They observed that individual site methylation did not change transcript levels after chronic restraint and concluded that there might be promoter-wide methylation changes. Desarnaud et al. applied a social defeat by exposing the model organism to another rat and inspected the promoter region of mice and found no increase in DNA methylation with the down-regulation of the Nr3c1 receptor in tissue samples from the hippocampus [37]. The inspection of a gene regulated by glucocorticoids, *ZBTB16* (involved in cellular migration and proliferation), demonstrated hypermethylation in response to prolonged exposure to glucocorticoids in human fetal lung fibroblast cells [38]. These results demonstrate the differences in methylation results for tissue sampling in model organisms such as rats, mice, and human fibroblast cells. 

A group of genes that code for specific proteins involved in the neuronal adhesion aspect of neuronal development, *NCAM* and *CHL1*, along with the serotonin receptor 5-hydroxytryptamine receptor 1A (5-HT1A), histone H3, and *OXTR* have been studied for changes of chronic stress. Desarnaud et al. also looked at the promoter regions for the genes, *NCAM* and *CHL1*, in mice that have been exposed to a rat or social defeat tests mimicking chronic stress from a social perspective [37]. This stress paradigm has been shown to induce social stress in mice via encountering an unfamiliar male in their home [39]. Interestingly, there was no DNA methylation change in the hippocampus between groups, suggesting that there was some other mechanism at play that down-regulated the adhesion molecules after chronic stress. When looking into the promoter site (-691 CpG) of 5-HT1A of male mice in the prefrontal cortex and midbrain, Le François et al. observed an increase in DNA methylation with an associated increase in 5-HT1A receptor after chronic unpredictable mild stress [40]. When chronic restraint stress was applied to adult male Sprague Dawley rats, Hunter et al. observed a mild increase in H3K4me3 and a reduction in H3K9me3 in the dentate gyrus [24]. An examination by Wiley et al. demonstrated that chronic stress by water avoidance altered proteins involved in tight junctions in the colon of Sprague Dawley rats via the up-regulation of IL-6 in association with H3K9 methylation and an increase in visceral hyperalgesia [41]. In an inspection of *OXTR* methylation patterns in relation to adult adversity (measured via Unmet Material Needs Scale and neighborhood crime) in (N = 100) African American women, Simons et al. observed increased methylation in the promoter region [42].

Surveying the effects of chronic stress on miRNAs surfaced two particular miRNAs, along with the gene responsible for the initiation of miRNA, *Drosha*. When (N = 35) adult male Long-Evans hooded rats underwent a 2-week restraint test, Babenko et al. discovered a down-regulation in miR-709 and up-regulation in miR-186 in the hippocampus and prefrontal cortex [43]. Computation analyses of targets for miR-186 were reported to be *Gabra4*, *Creb3*, *Eps15*, *A2bp1*, and *MAP3k2*, all important for various brain functions. For miR-709, the predicted targets were *Creb5*, *Efnb3*, *Nav1*, and *Nab1* and were found to be important for diverse brain functions. *Drosha*, an RNase Type III protein, was found to have decreased methylation in intron 9 in the dentate gyrus of (N = 121) adult male mice after 14 days of chronic social defeat [44]. Curiously, Hing et al. also observed increased methylation in the intergenic region of chromosome X after chronic social defeat stress. 

When looking at the effects of chronic stress on the heart, Zhang et al. examined different genes important for heart functioning [45]. In particular, they examined changes in desmin (forms the cytoskeleton in cardiac myocytes and aids in the mechanical strength of the heart), *Tgfb1* (associations with dilation in the left ventricle and dysfunction in the systole), and genes involved in the ASPC pathway that give sympathetic responsiveness in the heart in (N = 56) male mice. They showed that four consecutive weeks of the chronic restraint test had an associated increase in DNA methylation upstream of the gene that codes for desmin, which correlated with a decrease in desmin mRNA. Similar results were found in *Tgfb1* in the downstream region showing methylation and an associated decrease in mRNA levels. When examining the ASPC pathway genes involved in α1-adrenoceptor signaling such as *Ppp2r2c* (protein phosphatase 2, regulatory subunit B-γ), *Ppp2r1a* (protein phosphatase 2, regulatory subunit A), *Prkca* (protein Kinase C-α), and *Adra1b* (adrenergic receptor-α1B), Zhang et al. discovered that these genes showed alterations in DNA methylation that had associated changes of down-regulation in mRNA of *Adra1b* and *Ppp2r2c* while there was up-regulation of *Ppp2rla* and *Prkca*. 

Lastly, many studies have shown chronic stress can result in epigenetic imprints on genes whose functions have been classically traced to the stress response, such as *SLC6A4* or *BDNF*. Exploring the *BDNF* gene and its receptor, TrKB, Niknazar et al. observed an increase in the methylation of *BDNF* exon IV and TrKB in both male (N = 10) and female (N = 20) Wistar rats with an associated decrease in the expression of BDNF in the hippocampus after a forced swim test for 21 consecutive days [46]. Interestingly, females showed a higher methylation in *BDNF* when compared to male rats, potentially suggesting that female rats are more likely to be affected by chronic stress. Scientists assessing leukocytes in saliva observed lower DNA methylation in the *BDNF* CpG islands in exon 1 in the promoter region of Japanese workers (N = 774) with the highest job strain scores [47]. When chronic unpredicted mild stress was applied to rats, hippocampal *BDNF* (promoter region) demonstrated DNA hypermethylation in rats that also exhibited hyperhomocysteinemia [48]. This resulted in a reduction in the expression of the gene as well as cognitive decline. These results from *BDNF* demonstrate exon-specific variation in methylation in humans and animals with no supporting studies to validate findings. *SLC6A4*, a gene involved in the reuptake of serotonin, is another gene reported to have epigenetic changes as the result of high-stress environments, specifically in working female nurses (N = 49) [49]. Examining peripheral blood leukocytes revealed a decrease in methylation in the promoter region, potentially leading to an increase in the transcription of the reuptake receptor and less serotonin in the synaptic cleft. Alasaari et al. propose this as a potential coping mechanism for chronic stress. During an examination of chronic stress of living in disadvantaged neighborhoods, stress-related genes (*CRF* and *SLC6A4*) and inflammation-related genes (*F8* and *TLR1*) have altered methylation profiles in blood cell samples of B cells, T cells, neutrophils, and natural killer cells (N = 1226) [4]. Smith et al. showed that living in a neighborhood with socioeconomic disadvantages had DNA methylation in the non-promoter regions of *CRF*, *F8*, and *TLR1* and increased methylation in the shore/shelf site of *SLC6A4* [4]. The overall summary of these epigenetic changes associated with chronic stress can be seen in Table 2.

## 4. Early Childhood Stress

Unique to a specific developmental stage during one’s lifetime, trauma experienced early in life is usually referred to as an ACE (adverse childhood experience), ELE (early life stress), or CM (childhood maltreatment) in the research literature. Examples of early trauma experienced in childhood include physical, sexual, or emotional abuse or neglect, violence within the home, or a hostile social environment, i.e., bullying [50]. A multitude of studies suggests that early traumatic experiences are associated with changes to the epigenome that are also linked to defects in brain developmental programs, psychiatric diseases, and an increased risk for drug abuse and suicide. In addition to psychological disorders, childhood trauma is also significantly correlated with negative physical health outcomes such as an increased risk for metabolic syndromes, chronic pain, and cancer [51]. It has been shown that the molecular changes caused by such incidents of negative childhood experiences are dependent upon the genetics of the individual, the type of stress, and the timing of the stressful event such as that earlier stressful experiences may be associated with longer-term effects [52] (Miguel et al). Collectively, both animal and human studies in response to early life stress report changes in differentially methylated genic and intergenic regions of the genome, altered levels of stress response miRNAs, and different global methylation patterns compared to those individuals who have not experienced childhood trauma.

When investigating possible epigenetic changes associated with childhood stress, the results are mixed. For example, numerous studies have replicated the finding that childhood adversity is associated with an increase in the methylation of the *Nr3c1* promoter or the coding region of the gene in blood cells [53,54,55], but other studies show a decrease in the methylation of the *Nr3c1* promoter or coding region in blood [56,57] or no change [58]. One recent study examined the epigenetic effects of daily hassles (DH) or daily stress on the *Nr3c1* gene in adolescents. The results reported higher DNA methylation of *Nr3c1* in adolescents with higher levels of DH, and this was associated with blunted HPA axis reactivity to psychosocial stress. This research group also found a correlation between higher DH and longer HPA axis stress recovery. Additionally, participants with higher *Nr3c1* methylation had lower autonomic nervous system (ANS) adaptability to stress, as evidenced by lower parasympathetic withdrawal [59]. Another gene that has been shown in multiple studies to be epigenetically modified in response to childhood stress is the *FKBP5* gene. One research group reported the allele-specific *FKBP5* demethylation of intron 7 in blood cells [60], and another study showed decreased methylation [61] in leukocytes. On the contrary, a few other researchers show that there is no association between the methylation states of *FKBP5* and stress [58,62]. Similar to the conflicting results reported for *FKBP5*, epigenetic changes in *BDNF* (brain-derived neurotrophic factor) with childhood stress show the hypermethylation of either the *BDNF* promoter/coding region [63], hypomethylation [64], hypomethylation [64], or no association of methylation changes with stress [65]. *OXTR* (oxytocin receptor) gene changes have also been reported, to varying degrees. Hypermethylation in CpG sites in blood [66,67] was found to be associated with childhood trajectories of anxiousness, although other studies fail to show a link between childhood trauma and epigenetic changes in the *OXTR* gene [68,69].

Of all published studies in the literature examining the association of childhood trauma and epigenetic modifications with *SLC6A4* (serotonin transporter), the results suggest that there is an increase in methylation as a result of this type of stress in lymphoblasts [70,71], leukocytes [72], and blood [73]. Additionally, multiple studies show consistent changes in methylation globally across the genome [74] in the cingulate cortex and amygdala [60,75]. Other genes that show associations with childhood stress and epigenetic changes include *KITLG* [76], *PRDM14* [77], and *RAB14* [78]. Lastly, changes in miR-15a have been seen in blood cells from individuals who experienced the loss or separation of a parent, sexual abuse, or physical abuse [78].

Similar to results shown in humans, studies in rats reported early stress associated with increased methylation of *BDNF* [63,79]. Additional non-human studies looking at the epigenetic changes associated with early life stressors yielded hypomethylation in the *Nr3c1* of male mice [80], and a reduction in AVP methylation in mice hippocampi following maternal separation stress [81]. An interesting study in mice showed sex-dependent epigenetic changes associated with early life stress. Early life adversities induced a depressive state and altered miR-34a levels in adulthood under acute stress, but solely in females. Specifically in the dorsal raphe nuclei, this miRNA is associated with prefrontal-accumbal serotonin release under acute stress exposure in females. Additional genes modified following early stress in animal models showed changes in *MeCP2*, *CB1*, and *CRFR2* with maternal separation [82] and mi-RNA 133b when animal mothers were stressed during pregnancy [83]. Overall, studies do suggest that childhood stress does result in aberrations to the epigenome that persist throughout one’s lifetime and even through multiple generations (to be discussed later). The overall summary of these epigenetic changes associated with early childhood stress can be seen in Table 3.

## 5. Traumatic Stress

Traumatic stress is a form of stress that occurs in response to the rapid and often momentous loss of key valued resources [98]. Examples of trauma include natural disasters, war, sexual assault, tragic death, and other catastrophic events. Exposure to traumatic events has been recognized as part of the human experience and has the potential to impact subsequent development across the lifespan, although individual responses to trauma vary widely. Nevertheless, epigenetic changes have been reported in correlation with this type of stressful experience. Most studies report epigenetic changes for this type of stress in association with PTSD (post-traumatic stress disorder) or based on life experiences that are self-reported as traumatic events.

When comparing individuals diagnosed with PTSD to those non-PTSD control subjects, epigenetic changes abound. Hypomethylation is seen across the *Nr3c1* promoter [74,77,80,93], *UBE2L3* (Ubiquitin-conjugating enzyme) promoter [92], *AHRR* (aryl hydrocarbon receptor) [99], *F2R*, *CNPY2*, *BAIAP2L1*, and *TBXAS1* [100]. On the contrary, some research efforts have shown the hypermethylation of various gene regions. Traumatic events have shown a significant association with increased methylation levels of *CRHR1* [101], *MANC1* [102], *ADCYAP1R1* CpG island, and *PACAP* [103]. The gene *FKBP5* has also been shown to be associated with PTSD. Yehuda et al. propose a mechanistic model of the relationship between GR and *FKBP5* methylation in PTSD whereby GR responsiveness is increased resulting in a decrease in FKBP5 expression through a decrease in cortisol signaling. Additionally, epigenome-wide association studies or EWAS have revealed a number of genes epigenetically modified in correlation with PTSD severity. These genes include *BRSK1*, *LCN8*, *NFG*, *DOCK2*, *ZFP57*, and *RNF39* [102,103,104].

Significant DNA methylation changes in additional genes have also been seen in post-deployment soldiers with PTSD. These genes include *H19* and *IL18* [105]. Post-deployment cases of PTSD showed a decrease in methylation levels along the *H19* and *IL18* genes, resulting in increased levels of both of these proteins. *H19* codes for a long noncoding RNA thought to regulate body weight, cancer, inflammation, and aging [106]. Uddin et al. saw that two CpG island sites along the gene loci *NRG1* and *HGS* had increases in DNA methylation in PTSD combat veterans [102]. *NRG1* codes for Neuregulin-1 (*NRG1*), a component of the epidermal growth factor family, and induces the proliferation, differentiation, and survival of several cell types including epithelial cells, glial cells, neurons, and cardiomyocytes [107]. *HGS* is a gene that codes for hepatocyte growth factor-regulated tyrosine kinase substrate, which functions in regulating endosomal sorting and plays a critical role in the recycling and degradation of membrane receptors. The encoded protein sorts monoubiquitinated membrane proteins into the multivesicular body, targeting these proteins for lysosome-dependent degradation, and has been shown to play an important role in the central nervous system tissue [101]. Additionally, Montalvo-Ortiz et al. found that in 1135 male European–American U.S. veterans who participated in the National Health and Resilience in Veterans Study, CpG sites of genes involved in immune function, transcription regulation, axonal guidance, cell signaling, and protein binding were found to be differentially methylated. Among these, *SENP7*, which is involved in transcription regulation and has been linked to risk-taking behavior and alcohol consumption in genome-wide association studies, was replicated in an independent veteran cohort and was down-regulated in the medial orbitofrontal cortex of PTSD postmortem brain tissue. In a more recent study examining the blood cells of 290 trauma survivors, methylation analysis showed increases in the CpG site methylation of HPA-related genes, *POMC* and *CRHBP*, as predictors for chronic post-traumatic musculoskeletal pain. *POMC* is a gene that codes for proopiomelanocortin, a protein which is then cleaved to form functional peptides that play a role in the stress response, such as ACTH and melanocyte-simulating hormones [108]. 

Histone modification changes have also been seen in individuals with PTSD. A review by Zhang et al. concluded that histone modification that was enriched in the promoter regions of candidate genes such as the *BDNF* and *Cdk5* could significantly increase the risk of PTSD [109]. Alterations in levels of histone acetylation and methylation in the hippocampus, amygdala, and prefrontal cortex are associated with PTSD and play key roles in the consolidation, reconsolidation, and extinction of fear memory in PTSD-like animals. It is worth noting that histone modifications of genes in the stress response are mainly involved in the regulation of the immune system, the serotonergic system, the neuropeptide Y-ergic system, and NMDA receptor-related pathways. In addition, histone modification can be regulated by a variety of enzymes, leading to the flexible regulation of PTSD, making drugs that target histone modification good choices for the clinical treatment of PTSD. The overall summary of these epigenetic changes associated with traumatic stress can be seen in Table 4.

## 6. Suicide

Suicide completers are defined as those individuals who end their life suddenly, passing away from non-natural causes. Suicide non-completers are those observed to have thoughts of suicide or attempted suicide but did not end their life. Research in the area of suicide completers presents limitations in small sample sizes, the freshness of tissue, and being confined to post-mortem studies. Additionally, mimicking suicide conditions in other model organisms such as rats offers challenges to individuals in this field for obvious reasons. With these limitations, researchers have turned their focus to human subjects that have attempted suicide and rely on samples of harvested brain tissue from brain banks or blood samples. Often the suicide completers have other mental disorders such as major depressive disorder (MDD) or bipolar disorder and age differences that must be factored in when examining the data. 

One study examined epigenetic changes in suicide completers following a permutation test that controls for age since aging is associated with methylation changes [118]. Haghighi et al. demonstrated that the ventral prefrontal cortex, Brodmann area 47 (BA 47), of (N = 25) depressed-suicide completers had a substantial increase in DNA methylation in genes that involve embryonic and cellular development, cell cycle, cell death and survival, and behavior compared to (N = 28) control [118]. Examining the Brodmann area 10 of the prefrontal cortex in (N = 6) male suicide completers, Schneidera et al. observed lower global DNA methylation [119]. These results suggest region-specific epigenetic changes that occur in suicide brains post-mortem. 

Suicidal individuals compared to non-suicidal individuals have shown epigenetic changes in the genes *GRIK2*, *BEGAIN*, *BDNF*, and *TrkB*, all of which are involved in neuron survival, cell-to-cell communication, and development [120,121,122,123,124]. Intron 13 of *GRIK2* (glutamate ionotropic kainate receptor) and *BEGAIN* (brain-enriched granulated kinase-associated protein) were reported in the literature to be hypomethylated and hypermethylated, respectively, in MDD suicide patients’ (N = 76) cortical brain regions [120]. Looking at the hippocampus, Brodmann area 9, and the blood of suicide completers via hanging (N = 22), Ropret et al. found no methylation changes in the two brain regions and a decrease in methylation upstream of exon 1 in blood samples of the *BDNF* gene [121]. Interestingly, they also saw an increase in the transcription of *BDNF* (I-IX) in the Brodmann area 9 and not the hippocampus. When examining whole blood in a cohort of women with emotionally unstable personality disorder, Jamshidi et al. observed an increase in methylation in the promoter region of *BDNF* associated with suicide behaviors [125]. Turning our focus to the truncated version of the *BDNF* receptor (TrkB-T1), the 3′UTR region in BA 9 and 10 of (N = 11) suicide completers with low TrkB-T1 expression demonstrated epigenetic changes [122]. These epigenetic changes showed hypermethylation in the 3′UTR in suicide samples. Lastly, examining the *BDNF*-promoter region IV yielded hypermethylation of (N = 18) suicide individuals’ Wernicke areas with an associated down-regulation of *BDNF* [123,124]. Keller et al. also detected no correlation between TrkB or its truncated version TrkB-T1 and suicidal behaviors in the Wernicke area [123]. 

Another list of genes observed in the literature to have epigenetic changes in suicide brain samples includes *Elovl5*, *ARHGEF38*, *PSORS1C3*, *OXTR*, and *CYP2D6.* These genes are involved in the elongation of long-chain fatty acids, the regulation of catalytic activity, and a gene involved in psoriasis, respectively. In a blood sample study conducted on attempted suicide individuals with MDD (N = 22), the results showed *Elovl5* (elongation of very long chain fatty acids protein 5) upstream regions proximal to the transcription start site underwent DNA methylation, and downstream regions from the transcription start site underwent lower CpG methylation [126]. An examination in the prefrontal cortex (BA 46) of *ARHGEF38* (rho guanine nucleotide exchange factor 38) in (N = 23) suicide completers diagnosed with a form of bipolar disorder by the DSM-IV criteria demonstrated hypomethylation across four CpG sites [127]. Additionally, transcripts of *ARHGEF38* were decreased in brain tissue. A relatively new psoriasis susceptibility gene, *PSORS1C3* (psoriasis susceptibility 1 candidate 3), that lies close to genes involved in immune system regulation has surfaced in the literature with connections to MDD and suicide [128]. Murphy et al. saw hypomethylation in (N = 20) suicide completers with MDD in BA 11 and 25. An examination of the MT2 region of the *OXTR* (sites -901, -924, and -934) showed no correlation with suicidality assessed with the Columbia Suicide Severity Risk Scale Military Version in salivary samples of (N = 86) male Afghanistan and Iraq war veterans [129]. *CYP2D6*, a gene coding for the cytochrome P450 enzyme, demonstrated modifications of both hypo- and hypermethylation for males and females, respectively, and were correlated with suicidality, warranting further research [130].

Additionally, recent literature has shown a variety of different additional genes that have surfaced in relation to suicide. These genes and their protein products include *ZNF714*, *NRIP3*, *Nr3c1*, *5-HT1A*, *SKA2*, MAOA, *GABRA1*, and *CERC2*. Observations of *ZNF714* (zinc finger protein 714) and *NRIP3* (nuclear receptor interacting protein 3) were found to be hypomethylated and mixed methylation, respectively, in (N = 9) male hanging suicides BA 9 [131]. Additionally, Kouter et al. also observed a higher expression of both genes when compared to controls in the same study. Analysis of neuropsychiatric genes and protein products *Nr3c1*, *5-HT1A*, *SKA2* (spindle- and kinetochore-associated complex subunit 2), *MAOA* (monoamine oxidase A), *GABRA1* (gamma-aminobutyric acid type A receptor subunit alpha1), and *NRIP3* showed epigenetic changes in (N = 25) male suicide completers [132]. Specifically, *Nr3c1* in the prefrontal cortex and hippocampus demonstrated DNA methylation in the 5′UTR region for teenage suicide completers and an associated decrease in the expression of exon 1 [133]. A recent study by Kouter et al. found higher methylation in the 1B promoter region of *Nr3c1* in the insula and blood, whereas lower levels of methylation in the hippocampus and mixed results in the BA 46 and amygdala were detected. *SLC6A4_2* amplicon was hypomethylated in BA 46 while *5-HT1A* was hypomethylated in blood but hypermethylated in the insula for suicide completers. *SKA2* had mixed methylation data in all tissues examined. *MAOA* in BA 46 and insula were hypomethylated in amplicon *MAOA_2*. In the hippocampus and blood, *GABRA1* had decreased methylation while the insula had increased methylation of suicide individuals. Lastly, the *NRIP3* amplicon showed decreased methylation patterns in the hippocampus and insula [132]. Analysis of gene expression levels in suicide brain regions yielded a decrease in *SLC6A4* and *Nr3c1* gene products in the hippocampus, whereas *5-HT1A* showed a borderline increase [132]. A meta-analysis conducted by Zhu et al. showed a correlation of hypermethylation in *BDNF*, *SLC6A4*, and *Nr3c1* with a higher risk of depression [134]. An investigation of hypermethylation changes in the cerebellum conducted by Policicchio et al. showed one of the top-ranked suicide-associated genes as *CERC2*, a gene that has been shown to be involved in chromatin remodeling [135].

Lastly, new literature has surfaced reporting a new mouse model designed to study suicidality risk by increasing aggression in these mice through extended social isolation [136]. In particular, the researchers found that in mice subjected to social isolation for 4 weeks, there was an increase in aggression along with an increase in methylation and reduced expression of a gene known to be neuroprotective, *PPAR-α* [136]. The authors suggest that studying this mouse model may provide insight into mechanisms governing suicide risk associated with social isolation [136]. The overall summary of these epigenetic changes associated with suicide can be seen in Table 5.

## 7. Transgenerational Effects

Multiple studies have provided evidence that different types of stress exposure, and the associated epigenetic changes linked to maladaptive and poor mental health outcomes, can be passed down through generations [137,138,139]. Regarding chronic stress, Franklin et al. conducted a study with mice and showed that the effects of early chronic stress through changes in DNA methylation in the germline were transmitted through males and could affect the offspring in a sex-dependent manner [82]. In this study, mice were exposed to chronic and unpredictable maternal separation from days 1 to 14 following birth. The results showed the altered methylation of *MeCP2*, *CB1*, and the *CRFR2* CpG island in the first-generation (F1) germline and second-generation (F2) brain, along with decreased mRNA expression in F2 brain [82]. These changes in methylation were associated with depressive-like behaviors and, additionally, altered behavioral responses to novel and aversive environments in adult mice. Another study showed evidence of intergenerational stress through epigenetic changes. Pregnant Wistar rats received restraint stress during the last week of gestation with male offspring sacrificed on 28 days and 60 days following birth. Prenatal stress induced changes in gpm6a (neuronal membrane glycoprotein) levels in hippocampal and prefrontal cortex tissues and at both ages analyzed, indicating the persistence of this change over time [83]. Another study revealed that chronic stress was found to be transmitted through male Long-Evans rats who were stressed for 27 consecutive days and then mated with control female rats. Specifically at day 21, the offspring were sacrificed and global DNA methylation levels in the hippocampus and frontal cortex were analyzed. Paternal stress prior to conception altered the behavior of all offspring and male offspring specifically, and there was a noted reduction in stress reactivity to novel environments. Paternal stress also altered DNA methylation patterns in the offspring on day 21. Global methylation was reduced in the frontal cortex of female offspring but increased in the hippocampus of both male and female offspring [140]. Additionally, another study in female Wistar rats who were gavaged with 5 mg/kg of fluoxetine (Anti-depressant) FLX during early pregnancy and the last day of lactation found brain methylation changes in male rats. On postnatal day 75, global DNA methylation levels showed an increase in the hypothalamus, cortex, or PAG. Furthermore, early exposure to FLX was also associated with a reduction in time mice spent in social interaction and a decrease in the plasma corticosterone level during restraint stress. Altogether, this study showed results suggesting that maternal exposure to FLX during gestation and lactation results in a long-lasting impact on the DNA methylation of the hippocampus and affects social interaction HPA axis activity during unique types of stress [90]. 

Multiple studies suggest a possible transgenerational phenomenon associated with traumatic stress. In one study, the sample population consisted of 24 mothers and newborns in the eastern Democratic Republic of Congo, a region with extreme conflict and violence against women. Maternal experiences of war trauma and chronic stress were associated with *BDNF* methylation in umbilical cord blood, placental tissue, and maternal venous blood. The majority of significant associations were observed in transcription factor binding regions of *BDNF* [91]. In a separate study, researchers investigated genome-wide sperm DNA methylation patterns in trauma-exposed Vietnam veterans. At the genome-wide level, they identified three CpG sites associated with PTSD in sperm including two intergenic and one CpG within the *CCDC88C* gene. Of those associated with PTSD, in sperm, 1868 CpGs were also associated with PTSD in peripheral blood including the *RORA*, *CRHR1*, and *DOCK2* genes that have been previously implicated in PTSD. Additionally, 10 of these CpG sites were significantly associated with a reported history of a diagnosed mental health condition in children, with these same genes being reported to be resistant to demethylation, making them strong candidates for transgenerational inheritance. A different study found epigenetic effects based on the gene variant of interest. In mothers carrying the stress-sensitive T-allele for *FKBP5*, it was shown that maternal *FKBP5* methylation negatively correlated with threat-based ACEs (adverse childhood events) and maternal PTSD symptoms during pregnancy but not deprivation-based ACEs. In infants homozygous for the C allele, infant *FKBP5* methylation positively correlated with maternal threat-based ACEs and prenatal PTSD symptom severity, but not deprivation-based ACEs or adversity in adulthood, suggesting the type of ACE and allelic variant may affect the epigenetic change associated with symptoms of PTSD [81]. Lastly, Yehuda et al. found higher levels of methylation across the *FKBP5* gene in Holocaust survivors compared with controls and lower levels of *FKBP5* expression in offspring [112]. Altogether, these results suggest parental exposure to stress may be passed down to children, and the effects can persist over time.

## 8. Future Directions/Conclusions

In this review, we examined various epigenetic mechanisms seen with acute stress, chronic stress, early childhood stress, traumatic stress, and suicide. Upon examination of all these articles, we observed four particular genes that surfaced in all categories of stressors examined and suicide. These genes include *Nr3c1, OXTR, SLC6A4,* and *BDNF,* all of which demonstrated region- and site-specific methylation patterns in response to various stressors. 

In acute stress, numerous studies demonstrated general methylation in the *Nr3c1* gene in various regions and higher perceived stress reactivity, whereas the *BDNF* gene and promoter regional histone modification had no epigenetic changes [18,20,21,25,28]. Site-specific methylation patterns were observed in *OXTR* [28]. We were unable to find epigenetic alterations associated with *SLC6A4* in the literature for acute stress, indicating that acute stress may not be strong enough to elicit an epigenetic alteration in this gene. Other epigenetic modifications that were identified in multiple articles included a histone modification in various subunits and miR-124 in response to acute stressors [21,22,23,24,26].

When examining chronic stress, the literature demonstrated increased methylated changes in exon 1_7_ of *Nr3c1* in the amygdala (a fear-processing region) and no changes in the hippocampus or the pituitary and adrenal glands [34,36,37]. Examination of the *BDNF* gene epigenetic changes in response to chronic stress yielded an increase in methylation in exon IV and the promoter region of rats and the opposite for exon 1 in humans [46,47,48]. Chronic stress yielded increased methylation in the promoter and shore/shelf site of *OXTR* and *SLC6A4*, respectively [4,42]. Other epigenetic modifications that were identified in multiple literature articles included a histone H3 modification increase in methylation in K4/K9 (dentate gyrus/colonic tissue), whereas K9 showed a decrease in methylation (dentate gyrus) in response to chronic stress [24,41]. Interestingly, chronic water avoidance stress was associated with an up-regulation of the acute phase reaction promoter of *IL-6* [41].

In early childhood stress, the literature demonstrated mixed results for *Nr3c1* methylation in the promoter and coding regions [53,54,55,56,57,58]. *BDNF* gene also demonstrated conflicting results with childhood stress [63,64,65]. An examination of *OXTR* showed an association with anxiousness and hypermethylation in CpG sites [66,67]. However, some studies fail to demonstrate an epigenetic link with childhood stress [68,69]. *SLC6A4* has consistently been shown in the literature to have an increase in gene methylation in lymphoblasts, leukocytes, and blood with childhood stress [60,70,71,72,73,74,75].

Traumatic stress can cause epigenetic changes in the aforementioned genes as well. For *Nr3c1*, lower methylation in response to various traumatic stressors has been observed [70,73,76,86,88,105,128]. Opposite to *Nr3c1*, *BDNF* demonstrated hypermethylation in one combat veteran study in association with PTSD [114]. Exon region 3 of *OXTR* showed an increase in methylation for females with PTSD [109]. Lastly, the *SLC6A4* promoter region was examined in individuals with PTSD, and no association was unearthed [110]. Interestingly, Koenen et al. discovered that individuals with methylation in the promoter region of *SLC6A4* served as a protective effect against traumatic stress [117]. 

In suicide completers and non-completers, literature examining the insula and blood demonstrated *Nr3c1* to be more methylated in the 1B promoter region, whereas the hippocampus had lower methylation levels [132]. *BDNF* showed a decrease in methylation in suicide completers in exon 1 and hypermethylation in promoter region IV in the blood and Wernicke area, respectively [121,123]. In addition to this, the promoter region in women with emotionally unstable personality disorders demonstrated hypermethylation in blood samples [125]. The *OXTR* MT2 region showed no methylation associations with suicidality indicating that the thoughts of suicide may not be strong enough to elicit an epigenetic alteration in this gene [129]. Lastly, *SLC6A4* had hypermethylation in the amplicon region 2 with suicide completion for male human BA 46 [132]. 

**Figure 1:**
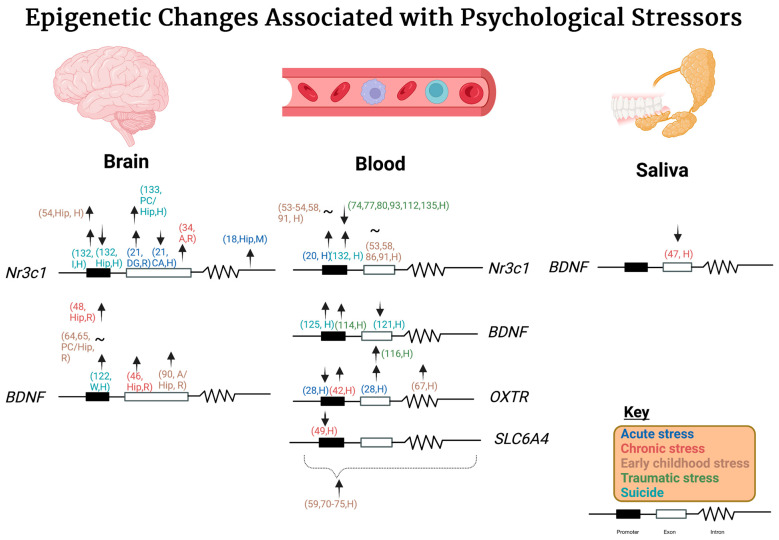
Stress-induced epigenetic changes associated with *Nr3c1*, *BDNF*, *OXTR*, and *FKBP5*. Stressors are color-coded with their respective epigenetic changes in the various tissue samples analyzed. Blue represents acute stress, red represents chronic stress, brown represents early childhood stress, green represents traumatic stress, and teal represents suicide. The simplistic gene diagram includes a promoter (blacked-out box), followed by an exon, (empty white box), and lastly by the intron (a squiggly line). Various abbreviations used include: H: humans, R: rats, M: mice, I: insula, Hip: hippocampus, Hi: hypothalamus, PC: prefrontal cortex, W: Wernicke’s area, A: amygdala, arrow up: increase in methylation, arrow down: decrease in methylation, tilde symbol: variable epigenetic changes, and numerical number corresponds to the source. The location of the arrow is not exact but will indicate whether the epigenetic change occurs in the promoter, intron, or exon region as shown in the key. An up arrow indicates increase in methylation and a down arrow indicates a decrease in methylation. If no methylation changes were observed the area was left blank. Created with BioRender.com (accessed on 12 March 2023).

Collectively, some of these studies have not been reproduced and specific results may only show up based on the model organism system, tissue sample location, or stressor. An overall summary of the specific epigenetic changes that occur in the *BDNF, Nr3c1, OXTR,* and *SLC6A4* genes with various types of stress can be seen in Figure 1. 

As outlined above, *Nr3c1*, *OXTR*, *SLC6A4*, and *BDNF* all vary in their associated epigenetic changes, depending on the types of associated stress exposures. Although further study is needed to investigate the full profile of epigenetic changes that occur following stress, current reported changes may provide insight into mechanisms governing development and susceptibility to stress-related pathologies such as PTSD or suicidality. Guide et al. demonstrated that allostatic load (i.e., cumulative stress) contributes to poor health outcomes including psychological dysfunction and depression [141]. Stress type, severity, and longevity likely contribute to a continuum between these epigenetic changes discussed. Specifically, research by Caradonna et al. demonstrated that even short-lived stress has a measurable impact on allostatic load [142]. Once we understand the complete profile of stress-induced epigenetic changes, preventative measures and actionable targets for treatment can be designed and implemented. 

When determining the significance of discovered stress-induced epigenetic changes based on published results, it is important to note the reproducibility of the result (or lack thereof), variation in sampling location, gene regions analyzed/reported, and the model organism used. These variations in epigenetic outcomes of different types of stress can be seen in the *Nr3c1* gene between rats and mice [34,36,37]. Additionally, there is potential for race-specific epigenomes, which have been shown to play a role in systemic diseases such as metabolic syndrome as outlined by Chitrala et al. [143]. Furthermore, there is a need for an increase in longitudinal human studies of both sexes and various races as these data are limited in the literature. Accessibility to more samples from humans and additional studies with large sample sizes will enable either validating or voiding animal data in terms of accuracy. Ultimately, this could offer better insight into the molecular underpinnings of the different types of stress and epigenetic changes, thus enabling the determination of accurate models that reflect the physiologic mechanism in humans. 

If various forms of stress cause specific epigenetic changes and these changes are associated with poor psychiatric and physiological health outcomes, targeting these marks for reversal offers hope to improve patient outcomes. Ferioli et al. reviewed the role of exercise in providing beneficial impacts for patients battling various stress-related pathologies such as cancer and neurodegenerative conditions via epigenetic mechanisms [144]. Additionally, Ieraci et al. demonstrated that physical exercise mimicked the acute stress response in the hippocampus of mice with an increase in mRNA levels of *BDNF* while preventing its decrease seen shortly after an acute stressor via histone H3 acetylation in the promoter region of *BDNF* [25]. Additionally, yoga has been shown to demonstrate some changes in DNA methylation patterns and proteins involved in immunity [145]. Coping mechanisms such as social support, avoidance, and problem-solving may not offer a way to prevent or alter the course of methylation caused by stress overload [146]. A pilot study conducted on veterans with PTSD showed that some psychotherapies may offer an epigenetic fix for methylation changes in *Nr3c1* and *FKBP5* genes; however, the results were not statistically significant and need further validation [147]. The use of specific psychotherapy such as narrative exposure therapy in patients with PTSD has demonstrated protective epigenetic effects in *Nr3c1* [148]. Additionally, a pilot study examining mechanisms of MDMA’s efficacy for treating PTSD found MDMA treatment-responsive patients showed more methylation change compared to placebo on one site of the *NR3C1* gene [149]. The results from both narrative exposure therapy and MDMA interventions illustrate the need for personalized medicine approaches to treating patients with trauma-related disorders. Additionally, Venditti et al. reviewed some of the recent literature highlights of meditation offering a way to prevent or reverse the effects that occur due to stress from the environment [150]. However, the researchers discuss the ambiguous nature of the molecular basis of meditation and if it acts on the same epigenetic locations or through another mechanism. This illustrates a potential gap in the literature. Collectively, this non-exhaustive list of potential non-pharmacological “fixes” may offer ways to inhibit and even reverse epigenetic changes that arise throughout a person’s life experiences. 

Epigenetics is still a relatively new area of research. The more we learn about stress-induced epigenetic changes, the better our chances are to develop preventative measures or treatments based on clinically actionable epigenetic-related targets so that we can treat and potentially break the devastating cycle of transgenerational stress. 

## Figures and Tables

**Table 1:** Summary of epigenetic changes to genes associated with acute stress.

	Gene	Epigenetic Changes	Stress Type	Method	Model Organism	Tissue Location
[16]	Whole-genome DNA methylation	Increase in methylation in concordance with increase in lymphocytes	Psychosocial stress	Illumina Infinium EPIC array	Human	Whole blood samples
[18]	3′UTR *Nr3c1*	Increase in 5-hmC	Acute restraint stress	Immunohistochemistry, tandem mass-spec, TAB-seq analysis	Male mice	Hippocampus
[20]	Promoter region 1-C of *Nr3c1*	Lower levels of methylation with lower stress reactivity, but failed to survive test corrections	Psychosocial stress	Methylation-sensitive polymerase chain reaction (PCR)	Human	Fasting blood samples
[21]	Upstream of exon 2 in *Nr3c1*/Region around exon I_7_ *Nr3c1*	No effect on CpG levels in both tissues/increase in methylation in dentate gyrus, decrease in Cornu Ammonis	Swim stress test	EZ 96-DNA methylation kit	Male Wistar rat	Dentate gyrus and Cornu Ammonis
[22]	Histone H3K9 at retro-transposable element loci	H3K9me3	Acute restraint stress	ChIP sequencing against H3K9me3	Male Sprague Dawley rats	Hippocampus
[23]	Histone H3K7	H3K27me3	Acute heat stress	Nano-UPLC-ESI-Q-TOF-MS/MS	Male Taiwan country chickens	Adrenal gland
[24]	Histone H3K9/H3K27	Increase in H3K9me3 and decrease in H3K9me1 in both tissues/reduction in H3K27 me3 in both tissues	Acute restraint stress	Immunohistochemistry analysis	Adult male Sprague Dawley rats	Dentate gyrus and CA1
[25]	Histone H3 in promoter region of *BDNF*	No methylation or acetylation change	Acute restraint stress	ChIP assay	Male mice	Hippocampus
[21]	MiR-124a	Increased expression	Forced swim test	MicroRNA analysis targeted against miR-124a	Male Wistar rats	Dentate gyrus
[26]	MiR-135a/miR-124	Down-regulated	Acute restraint stress	Microarray analysis and reverse transcription real-time PCR	Adult male mice	Amygdala
[27]	*CYP24A1, BRCA2, NOTCH2, FOXO3, GATA3*	Methylated	Acute exposure to UV light	Infinity methylation nEPIC array	Female Caucasian humans	Skin from lower back
[27]	*KRT17, CSNK2A2*	Hypomethylated	Acute exposure to UV light	Infinity methylation nEPIC array	Female Caucasian humans	Skin from lower back
[27]	*CARD14*/*IRF8*	Demethylated/hypermethylated	Acute exposure to UV light	Infinity methylation nEPIC array	Female Caucasian humans	Skin from lower back
[28]	“*OXTR1*”/“*OXTR2*”/*BDNF* exon V, Vh, and Vi	Increase in methylation/decrease in methylation/no methylation differences	TSST	Sequenom EpiTYPER	Human	Blood samples
[29]	*PRF1* CpG sites -776 and -774	Increase in methylation	TSST	Quantitative methylation analysis by Bisulfite-pyrosequencing	Chronic fatigue syndrome humans	Blood samples
[19]	*Enpp2*/*Sostdc1*	Hyper-DhMR	Acute restraint stress	Genome-wide map of 5-hmC via NEBNext ChIP-Seq and RNAseq	Mice	Hippocampus
[19]	*Ulk4*/*Wnt9a*	Hypo-DhMR	Acute restraint stress	Genome-wide map of 5-hmC via NEBNext ChIP-Seq and RNAseq	Mice	Hippocampus
[19]	*Banp*/*Gadd45b*/*Cbfa2t3*/*Irs2*/*Klf15*/*Smtn*/*Spns2*	Hypo-DhMR	Acute restraint stress	Genome-wide map of 5-hmC via NEBNext ChIP-Seq and RNAseq	Mice	Hippocampus

**Table 2:** Summary of epigenetic changes to genes associated with chronic stress.

	Gene	Epigenetic Changes	Stress Type	Method	Model Organism	Tissue Location
[34]	*Nr3c1* promoter region of exon 1_7_	Increase in DNA methylation	Chronic water avoidance stress test (7 days)	Sodium Bisulfite sequencing, pyrosequencing	Female Fischer-344 rats	Central nucleus of the amygdala
[36]	*Nr3c1* promoter region of exon 1_7_	No individual site methylation change	Chronic restraint test (1 h)	Bisulfite sequencing	Male Sprague Dawley rats	Adrenal and pituitary gland
[37]	*Nr3c1* promoter region	No increase in DNA methylation	Social defeat test by rat exposure	Sodium Bisulfite sequencing	Mice	Hippocampus
[37]	*NCAM*/*CHL1*	No DNA methylation changes	Social defeat test by rat exposure	Sodium Bisulfite sequencing	Mice	Hippocampus
[38]	*ZBTB16*	Hypermethylation	Prolonged exposure to glucocorticoids	GenFind V3 DNA extraction and purification kit, Illumina Infinium HumanMethylationEPIC BeadChip	Humans	Fetal lung fibroblast cells
[40]	5-HT1A promoter (-691 CpG)	Increase in DNA methylation	Chronic unpredictable mild stress	Bisulfite treated, PCR, followed by random selection of amplifications and DNA methylation sequencing	Male mice	Prefrontal cortex and midbrain
[24]	Histone H3	H3K4me3 mild increase, H3K9me3 decrease	Chronic restraint test (7 days)	Immunohistochemistry analysis	Adult male Sprague Dawley rats	Dentate gyrus
[41]	Histone H3	H3K9 methylation increase, IL-6 up-regulation	Chronic water avoidance stress (10 consecutive days)	Chip, quantitative PCR, Western blot, and immunofluorescence	Young-adult male Sprague Dawley rats	Colonic
[42]	*OXTR* promoter region	Increased DNA methylation	Adult adversity (measured via Unmet Material Needs Scale and neighborhood crime)	Illumina 450K Human Methylation BeadChip	African American Women	Blood
[43]	MiR-709/miR-186	Down-regulation/up-regulation	Chronic restraint test (2 weeks)	miRNA microarray analysis using µParaflo ® Biochip	Adult male Long-Evans hooded rats	Hippocampus and prefrontal cortex
[44]	*Drosha* intron 9/chromosome X intergenic region	Decrease methylation/increased methylation	Chronic social defeat (14 days)	Methyl-Seq followed by Bisulfite-pyrosequencing	Adult male mice	Dentate gyrus
[45]	Desmin upstream region/*Tgfb1* downstream region	Increase in DNA methylation/increase in DNA methylation	Chronic restraint test (4 weeks)	Reduced representation Bisulfite sequencing and methylation-specific PCR	Male mice	Heart
[45]	*Ppp2r2c*/*Ppp2r1a*/*Prkca*/*Adra1b*	Alterations in DNA methylation	Chronic restraint test (4 weeks)	Reduced representation Bisulfite sequencing and methylation-specific PCR	Male mice	Heart
[46]	BDNF exon IV/TrkB	Increased methylation/increased methylation	Forced swim test (21 days)	One-way ANOVA, real-time PCR, and Western blotting	Male and female Wistar rats	Hippocampus
[47]	*BDNF* CpG islands in exon 1 promoter region	Lower DNA methylation	Chronic high job stress	Illumina Infinium HumanMethylation 450 BeadChip array	Japanese workers	Leukocytes in saliva
[48]	*BDNF* promoter region	DNA hypermethylation in rats with hyperhomocysteinemia	Chronic unpredicted mild stress	Methyl-Specific PCR, Sequenom Mass Spectrometry, and enzyme-linked immunosorbent assay	Rats	Hippocampus
[49]	*SLC6A4* promoter region	Decrease in methylation	Chronic job stress	Methylation 450K BeadChip and Bisulfite sequencing	Female nurses	Peripheral blood leukocytes
[4]	Non-promoter *CRF*/shore shelf site of *SLC6A4*	DNA methylation/increased methylation	Chronic stress from living in disadvantaged neighborhoods	Illumina HumanMethylation450 BeadChip	Humans	B cells, T cells, Neutrophils, and natural killer cells found in blood
[4]	Non-promoter *F8*/non-promoter *TLR1*	DNA methylation	Chronic stress from living in disadvantaged neighborhoods	Illumina HumanMethylation450 BeadChip	Humans	B cells, T cells, Neutrophils, and natural killer cells found in blood

**Table 3:** Summary of epigenetic changes to genes associated with early childhood stress.

	Gene	Epigenetic Changes	Stress Type	Method	Model Organism	Tissue Location
[84]	*FRMD4A*, *CCDC174*, *FBXL2*, *CHD4*	Methylation status associated with cortisol levels	Torture and/or sexual assault	Epigenome-wide methylation via illuminates Infinium HumanMethylationEPIC BeadChip	Human mothers	Blood
[82]	*MeCP2*, *CB1*, *CRFR2*	Methylation of the CpG island surrounding the transcription initiation site of MeCP2 and CB1 genes was increased/CRFR2 gene methylation in a stretch of the CpG island located 5′ of the transcription initiation site was decreased	Chronic and unpredictable maternal separation from postnatal days 1–14	Bisulfite-converted DNA followed by pyrosequencing analysis	Mice	Germline
[76]	*KITLG* (cg27512205)	Methylation	Childhood trauma	Genome-wide analysis	Healthy humans	Blood and buccal cells
[77]	*PRDM14*	Hypomethylated	Childhood chronic stress assessed via hair cortisol	Hair cortisol levels in relationship to whole-genome DNA-methylation sequencing	5-year-old children	Blood and hair cortisol
[83]	*gpm6a* intron 1/miRNA-133b	No overall differences in methylation, CpG 6 methylation, and CpG 7 not methylated/overexpression of miRNA suppressed gpm6a mRNA	Restraint test on mothers in last week of gestation	Bisulfite conversion and quantitative PCR	Male offspring mice (postnatal days 28 and 60)	Hippocampus and prefrontal cortex
[85]	miRNA-34a	Increased	Maternal separation followed by repeated cross-fostering	Quantitative real-time PCR	Female mice	Medial prefrontal cortex and dorsal raphe nuclei
[80]	*Nr3c1* CpG island shore region	Hypomethylation	Maternal separation	Bisulfite sequencing	Male mice (postnatal day 0)	Hypothalamic neurons
[86]	*FKBP5*	Methylation with CC gene	Adverse childhood experiences	Bisulfite pyrosequencing and DNA methylation analysis	Postpartum women and babies (within 24 h)	Saliva
[58]	*Nr3c1*, *FKBP5*, *BDNF*, *AVP*, *CRHR1*, *SLC6A4*	No robust epigenetic variation	Physical, emotional, or sexual abuse/neglect/intimate-partner violence/crime/bullying/cyber-victimization	Bisulfite sequencing with Illumina Infinium HumanMethylation450 BeadChip	Human twin cohort (assessed at ages 5, 7, 10, 12, and 18)	Peripheral blood
[87]	*RAB14*	Decrease in methylation	Child bullying	Epigenome-wide methylation, Bisulfite conversion followed with Illumina Infinium HumanMethylation450 BeadChip	Children	Blood
[88]	*Nr3c1* amplicon 1	Higher methylation	Perinatal stress/stressful life events/traumatic youth experiences	Bisulfite converted DNA, PCR, reverse transcription, cleavage of RNA product followed with mass spectrometry	Children (mean age 16.1)	Blood
[89]	*Nr3c2*, *Nrxn1*, *Nfia*, and *Clip1*	Variable methylation in adult female mice who had experienced early life stress	Early life stress before weaning (postnatal days 12 to 18)	Chemical labeling-based 5hmC enrichment	Female mice 3-month-old	Hypothalamus
[79]	*BDNF* exon IV	Increase methylation	Prenatal stress	Bisulfite pyrosequencing genomic DNA	Male offspring of pregnant Sprague Dawley rats	Amygdala and hippocampus
[90]	Global DNA methylation	Increase in DNA methylation in hippocampus	Medication used for antidepressants	Imprint methylated DNA quantification Kit	Female Wistar rats	Hippocampus, cortex, hypothalamus, and periaqueductal gray matter
[91]	*BDNF*	Increase in DNA methylation with trauma exposure	War trauma	HumanMethylation450 BeadChip	Mothers and newborns in the eastern democratic republic of Congo	Umbilical cord blood, placental tissue, maternal venous blood
[53]	*Nr3c1*	Increase in DNA methylation	Early childhood abuse	Bi-sulfite sequencing	Women from Black Women’s Health Society	leukocytes
[54]	*Nr3c1*	Increase in promoter methylation	Early childhood abuse	Bi-sulfite sequencing	Post-mortem suicide victims of childhood abuse	Hippocampus sections post-mortem
[92]	*Nr3c1*	Increase in DNA methylation	Early childhood physical abuse	Bi-sulfite sequencing	Subjects with Borderline Personality Disorder	Peripheral Blood Cells
[55]	*Nr3c1*	Increase in promoter methylation	Lack of adequate nurturing, as measured by parental loss, childhood maltreatment, and parental care	Bi-sulfite sequencing	Healthy adults	Leukocytes
[93]	*Nr3c1*	Lower *Nr3c1* methylation	Schizophrenia patients with ACES	Pyrosequencing	Human	Leukocytes
[59]	*Nr3c1* CpG sites	Increased methylation	TSST for an acute stress	Pyrosequencing	Human	Saliva
[60]	*FKBP5*	Demethylation in functional glucocorticoid response elements of FKBP5 gene	Childhood trauma	Bisulfite pyrosequencing	Human	Whole blood cells
[94]	*LINE1*	Lower LINE1 methylation	Schizophrenia patients with ACES	Pyrosequencing	Human	Whole blood leukocytes
[61]	*FKBP5*	Decreased levels of DNA methylation	mother-infant dyads shortly after parturition	Mass array spectrometry	Human	Immune cells from blood
[56]	*FKBP5*	ACES associated with lower methylation levels at CpG site	Patients with psychotic disorders (with and without ACES)	Pyrosequencing	Human	Peripheral Blood leukocytes
[63]	*BDNF*	Hypermethylation of promoter	Maltreatment compared to positive caregiving	Methylation-specific real-time PCR (MSP) or direct bisulfite DNA sequencing PCR (BSP) on bisulfite-modified DNA (Chemicon or Qiagen), or via methylated DNA immunoprecipitation using an antibody against 5-methylcytosine	Rats	Prefrontal cortex and hippocampus
[95]	*BDNF* promoter I and IV	No long-term epigenetic changes	Early life abuse, experiencing a war or natural disaster, and poverty	Sequenom MassArray	Humans	Blood and buccal tissue
[66]	*OXTR*	Hypermethylation of CpG sites predictive for pathology	393 African American adults with or without childhood abuse	Bisulfite sequencing	Human	Whole blood
[67]	*OXTR*	ELA exposure was associated with one significant CpG site in the first intron among females, but not among males	46 adults (23 males/23 females) with varying degrees of childhood adverse events	Pyrosequencing	Human	Whole blood
[70]	*SLC64A*	Significant effect of Child Abuse on overall methylation levels	192 (96 males, 96 females from Iowa Adoption Study) with varying levels of childhood abuse experiences	Bisulfite conversion	Human	lymphoblasts
[71]	*SLC64A*	Examination of these four CpG residues indicated that methylation of cg22584138 was influenced by both genotype and sex abuse, whereas methylation of cg05016953 was influenced only by sex abuse history	158 female subjects in the Iowa Adoption Studies	Illumina HumanMethylation450 BeadChip	Human	lymphoblasts
[72]	*SLC64A*	Higher promoter methylation status was significantly associated with childhood adversities	108 patients with major depressive disorders	Bisulfite sequencing	Human	Leukocytes
[73]	*SLC64A*	Childhood trauma, being male, and smaller hippocampal volume were independently associated with greater peripheral serotonin transporter methylation	Thirty-three adults with Major Depressive Disorder (MDD) (23 females) and 36 matched healthy controls (21 females) were included in the study	Pyrosequencing	Humans	Whole Blood
[74]	Whole genome	362 differentially methylated promoters in individuals with a history of abuse compared with controls. Among these promoters, 248 showed hypermethylation and 114 demonstrated hypomethylation, highest methylation difference in *ALS2* gene	41 French-Canadian men (25 with a history of severe childhood abuse and 16 control subjects)	Methylated DNA immunoprecipitation (meDIP) method	Humans	Cingulate cortex
[75]	Whole genome	A history of child abuse was associated with cell type-specific changes in DNA methylation of oligodendrocyte genes and a global impairment of the myelin-related transcriptional program	Postmortem brain samples from human subjects (N = 78) and from a rodent model of the impact of early life environment (N = 24) were analyzed	Bisulfite sequencing	Humans	Amygdala/Post-mortem brains
[60]	Whole genome	Genome-wide methylation evidence of distinct biological modifications in PTSD in the presence or absence of exposure to childhood abuse	396 with 169 trauma-exposed individuals	HumanMethylation 450k BeadChip	Humans	Whole Blood
[76]	*KIT4G*	Nine DMRs replicated across cohorts, respectively associated with the ACE score	Two cohorts (mothers from the Avon Longitudinal Study of Parents and Children, ALSPAC, *n* = 780 and women from the MRC National Survey of Health and Development, NSHD, *n* = 552	HumanMethylation450 BeadChip	Humans	Peripheral Blood
[87]	*RAB14*	One site, cg17312179, showed small changes in DNA methylation associated with bullying exposure and RAB14 methylation levels decreased for exposed but increased for nonexposed	Population-based Generation R Study and Avon Longitudinal Study of Parents and Children (combined *n* = 1352)	Bisulfite sequencing	Humans	Peripheral Blood
[78]	mIR-15a	Increased levels of childhood stress	Childhood adversity	Affymetrix miRNA 2.0 array	Humans	Blood cells
[81]	*AVP*	Decrease methylation	Maternal separation	Bisulfite sequencing	Mice	hippocampus
[96]	*Nr3c1*	CM was associated with an increase in DNA methylation in an EGR1 transcription factor binding site	Childhood maltreatment (CM)	Pyrosequencing	147 adult participants from the Detroit Neighborhood Health Study	Whole blood
[97]	*FKBP5*	No change in methylation for childhood abuse	Childhood abuse	Pyrosequencing	3965 subjects of the Study of Health in Pomerania	Whole blood
[57]	*Nr3c1*	Associations between DNA methylation and severity of fatigue as well as with childhood emotional abuse in CFS patients, although these findings were not significant after correction for multiple testing	Childhood trauma	Bisulfite sequencing	80 female CFS (chronic fatigue syndrome) patients and 91 female controls	Peripheral blood
[69]	*OXTR*	Methylation of both OXTR and OXT genes shaped the directionality of adversity effects	Memories of Childhood Trauma	Bisulfite sequencing	81 women	Blood
[65]	*BDNF*, *Nr3c1*, and *MAN2C1*	Increased methylation with adverse childhood experiences	Adverse childhood experience [ACE] score	Pyrosequencing	70 active military members with and without PTSD	Peripheral blood

**Table 4:** Summary of epigenetic changes to genes associated with traumatic stress.

	Gene	Epigenetic Changes	Stress Type	Method	Model Organism	Tissue Location
[100]	*F2R*, *CNPY2*, *BAIAP2L1*, *TBXAS1*	All sites showed lower DNA methylation	PTSD severity	MethylationEPIC BeadChip	PTSD military personnel	Blood
[92]	*UBE2L3*	Differentials methylated and promoter was hypomethylated in PTSD and MDD patients	Traumatic event	Bisulfite sequencing	PTSD with MDD, MDD alone, and no PTSD or MDD	Peripheral blood
[101]	*CRHR1*	Increase in methylation from baseline in rs110402 GG allele individuals after treatment	Traumatic event	Illumina 450K array and Bisulfite conversion	Women with PTSD	Blood
[110]	Epigenetic age	Traumatic stress associated with advanced epigenetic age	Traumatic event	Illumina Infinium HumanMethylation BeadChip	Humans	Peripheral blood
[102]	*MAN2C1*	Higher methylation had increased risk of PTSD	Traumatic event	Bisulfite conversion followed by Infinium HumanMethylation 27K BeadChip	Humans from Detroit	Blood
[104]	*ZFP57*, *RNF39*, *HIST1H2APS2*	Decrease in DNA methylation with increased PTSD symptoms	Combat trauma leading to PTSD	Illumina HumanMethylation 450 BeadChip	Dutch military cohort and male US marine cohort	Blood
[74]	*Nr3c1* promoter regions 1B and 1C	Lower methylation levels	PTSD from trauma	Sequence EpiTYPER, Sodium Bisulfite conversion	Humans with PTSD	T-lymphocyte isolated from blood
[99]	*GOS2* cg19534438/*AHRR* cg05575921	Methylated at locus/decreased methylation at locus	PTSD from trauma	EWAS using Illumina EPIC methylation BeadChip	Humans with PTSD	Blood samples and prefrontal cortex
[105]	H19 and IL18	Those who did not develop PTSD had reduced %5-mC levels of H19 and IL18 after deployment	PTSD from combat trauma	Bisulfite treatment followed by pyrosequencing	Post-deployment military individuals with PTSD	Blood serum
[111]	*HEXDC* rs4789774/*MAD1L1*	Development of combat-related PTSD is associated with distinct methylation patterns in HLA region, HEXDC, and MAD1L1	PTSD from combat trauma	HumanMethylation450 BeadChip	Three cohorts of male military members	Blood
[91]	*CRH*, *CRHBP*, *Nr3c1*, and *FKBP5*	Methylation changes in offspring associated with war exposure in mothers	War trauma	HumanMethylation450 BeadChip	Mothers and newborns in the eastern Democratic Republic of Congo	Umbilical cord blood, placental tissue, maternal venous blood
[4]	*AHRR*	Lower DNA methylation in PTSD	Traumatic event	Illumina HumanMethylation450 BeadChip	Military and civilian cohorts	Blood
[60]	*SENP7*	Six genome-wide significant (GWS) CpG sites associated with past-month PTSD and three CpGs with lifetime PTSD	PTSD from the Vietnam War	Bisulfite converted analysis	1135 male European–American U.S. veterans who participated in the National Health and Resilience in Veterans Study (NHRVS)	Semen and blood
[112]	*Nr3c1* 1_F_ promoter	Lower methylation in veterans with PTSD	PTSD from combat trauma	Bisulfite mapping and colonial sequencing	Male combat veterans	Peripheral blood mononuclear cells
[113]	H3K4me3 histone modification, WNT 10B	Increase in H3K4me3 around WNT 10B promoter in patients with PTSD	PTSD from various traumatic stressors	RNA-seq, ChIP-seq, and microarray	Patients with PTSD	Peripheral blood mononuclear cells
[102]	*NRG1*, *HGS*	Two CpG sites significantly associated with current PTSD in NRG1 (cg23637605) and in HGS (cg19577098)	PTSD from combat trauma	Methylation microarray	Patients with PTSD	Whole blood cells
[103]	5600 CpG islands	Majority of CpG islands were hypermethylated in PTSD cases	PTSD from combat trauma	Agilent whole genome methylation array/targeted bisulfite sequencing	Operation Enduring Freedom/Iraqi Freedom Combat veterans	Peripheral whole blood cells
[114]	*BDNF*	Subjects with PTSD showed a higher DNA methylation of four CpG sites at the BDNF promoter compared with those without PTSD	Combat veterans with PTSD	Pyrosequencing	Combat veterans	Peripheral blood cells
[115]	*ADCYAP1*	Methylation of ADCYAP1R1 is associated with PTSD	Patients with PTSD	HumanMethylation27 BeadChip	Patients receiving services in the primary care clinics at Grady Memorial Hospital	Whole blood
[116]	*OXTR* exon 3	CpG islands increase in methylation in female PTSD	PTSD	Bisulfite converted analysis	67 human subjects (31 PTSD, 36 controls)	Whole blood
[117]	*SLC6A4* promoter	No association, increase in methylation demonstrated protective effects	PTSD	HM27 BeadChip	100 human subjects	Whole blood
[108]	*POMC* and *CRHBP*	CpG sites demonstrated increased methylation as predictor for chronic post-traumatic musculoskeletal pain	PTSD	Bisulfite conversion and Illumina Infinium Human MethylationEPIC BeadChip array	Humans	Blood

**Table 5:** Summary of epigenetic changes to genes associated with suicide.

	Gene	Epigenetic Changes	Stress Type	Method	Model Organism	Tissue Location
[120]	*GRIK2* intron 13/*BEGAIN*	Hypomethylated/increase in methylation	Suicide completion w/MDD	Genome-wide methylation sequencing via MBD-Seq	Humans	Cortical brain region
[121]	*BDNF* exon 1	Decrease in methylation	Suicide completion via hanging	Bisulfite next-generation sequencing	Humans	Blood
[122]	3′UTR TrkB-T1	Hypermethylation	Suicide completion with low TrkB-T1	Methylated DNA immunoprecipitation, labeling, and hybridization via microarray	Humans	BA 8 and 9
[123]	*BDNF* promoter region IV	Hypermethylation	Suicide completion	MassArray methylation analysis	Humans	Wernicke area
[125]	*BDNF* promoter	Increased methylation	Suicidal patients with emotionally unstable personality disorder	Illumine EPIC BeadChip	Humans (women)	Blood
[126]	*Elovl5* upstream/downstream to transcription start site	Methylation/lower CpG methylation	Suicide non-completers with MDD	Bisulfite pyrosequencing	Humans	Blood
[127]	*ARHGEF38*	Hypermethylation across 4 CpG sites	Bipolar disorder suicide completion	SureSelect(XT) system, methyl-Seq, confirmation via pyrosequencing	Humans	BA 46
[128]	*PSORS1C3*	Hypomethylation	Suicide completion w/MDD	Bisulfite conversion followed by Infinium HumanMethylation450 BeadChip Array, then Bisulfite pyrosequencing confirmation	Humans	BA 11 and 25
[129]	*OXTR* MT2 region (-901, -924, -934)	No methylation at sites	Suicidality	Methylation assay via Bisulfite conversion followed by pyrosequencing	Humans (male veterans)	Saliva
[130]	*CYP2D6* CpG sites	Hypomethylation (males) and hypermethylation (females)	Severe suicide behavior	Illumina Infinium Methylation EPIC BeadChip	Humans	Peripheral blood
[131]	*ZNF714*/*NRIP3*	Hypomethylated/mixed methylation	Suicide completion via hanging	Next-generation sequencing of genome-wide methylation analysis	Humans	BA 9
[132]	*Nr3c1* 1B promoter	Higher methylation in the insula and blood, lower levels of methylation in hippocampus	Suicide completion	Bisulfite next-generation sequencing	Male Humans	Insula, blood, and hippocampus
[133]	*Nr3c1* 5′UTR	DNA methylation	Suicide completion	DNA methylation enrichment assay	Teenage humans	Hippocampus and prefrontal cortex
[132]	*SLC6A4*_2 amplicon	Hypermethylated	Suicide completion	Bisulfite next-generation sequencing	Male Humans	BA 46
[132]	*5-HT1A*	Hypomethylated in blood/hypermethylated in insula	Suicide completion	Bisulfite next-generation sequencing	Male Humans	Blood and insula
[132]	*SKA2*	Mixed methylation data in all tissues	Suicide completion	Bisulfite next-generation sequencing	Male Humans	Hippocampus, insula, amygdala, BA 46, and blood
[132]	MAOA_2 amplicon	Hypomethylated in insula and BA 46	Suicide completion	Bisulfite next-generation sequencing	Male Humans	Insula and BA 46
[132]	*GABRA1*	Decreased methylation in hippocampus and blood/increased methylation in insula	Suicide completion	Bisulfite next-generation sequencing	Male Humans	Hippocampus, blood, insula
[132]	*NRIP3* amplicon	Decrease methylation	Suicide completion	Bisulfite next-generation sequencing	Male Humans	Hippocampus and insula
[135]	*CERC2* intronic region	Hypermethylation across 4 CpG sites	Suicide completers	Illumina HumanMethylation450K BeadChip or Infinium MethylationEPIC BeadChip	Human	Cerebellum
[136]	*PPAR-* *α*	Increased methylation in CpG regions	Social isolation (4 weeks) model for suicide-like behavior/PTSD	Methyl-DNA immunoprecipitation	Male Swiss-webster mice	Hippocampus

## Data Availability

Pubmed-NIH and other various widely accessible and trusted search engines.
